# Ultraflexible Sensor Development via 4D Printing: Enhanced Sensitivity to Strain, Temperature, and Magnetic Fields

**DOI:** 10.1002/advs.202411584

**Published:** 2024-12-24

**Authors:** Yanbei Hou, Hancen Zhang, Kun Zhou

**Affiliations:** ^1^ Singapore Centre for 3D Printing School of Mechanical and Aerospace Engineering Nanyang Technological University Singapore 639798 Singapore; ^2^ Environmental Process Modeling Centre Nanyang Environment and Water Research Institute Nanyang Technological University Singapore 639798 Singapore

**Keywords:** 4D printing, magnetic force‐driven actuator, strain sensor, temperature sensitivity

## Abstract

This paper addresses the trade‐off between sensitivity and sensing range in strain sensors, while introducing additional functionalities through an innovative 4D printing approach. The resulting ultraflexible sensor integrates carbon nanotubes/liquid metal hybrids and iron powders within an Ecoflex matrix. The optimization of this composition enables the creation of an uncured resin ideal for Direct Ink Writing (DIW) and a cured sensor with exceptional electromechanical, thermal, and magnetic performance. Notably, the sensor achieves a wide linear strain range of 350% and maintains a stable Gauge Factor of 19.8, offering an ultralow detection limit of 0.1% strain and a rapid 83‐ms response time. Beyond superior strain sensing capabilities, the sensor exhibits outstanding thermal endurance for temperatures exceeding 300 °C, enhanced thermal conductivity, and a consistent resistance‐temperature relationship, making it well‐suited for high‐temperature applications. Moreover, the inclusion of iron particles provides magnetic responsiveness, enabling synergistic applications in location and speed detection, particularly in home care. Leveraging DIW facilitates the creation of complex‐shaped sensors with multiple functional materials, significantly broadening the sensor's capabilities. This convergence of additive manufacturing and multifunctional materials marks a transformative step in advancing the performance of next‐generation sensors across diverse domains.

## Graphical abstract

A successful formulation of conductive ink is prepared for direct ink writing. Through precise control of the ink composition, ultraflexible strain sensors with exceptional electromechanical, thermal, and magnetic performance are developed. The sensors have potential applications in human/robotic motion recording and health/temperature/magnetic field monitoring. This adaptability opens up opportunities for advanced wearable devices that require real‐time sensing across multiple parameters.

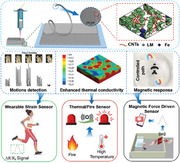

## 1 Introduction

In recent years, remarkable advances have been achieved in the fields of wearable electronics and artificial intelligence (AI).^[^ ^1^, ^2^, ^3^, ^4^, ^5^ ^]^ Wearable electronic devices collect data from human activities, while AI analyzes this data to monitor health and sports activities. Within the domain of wearable electronics, particular attention has been drawn to wearable and flexible strain sensors.^[^ ^6^ ^]^ These sensors have attracted significant interest due to their ability to detect various human activities, including joint motion, breathing, and vocalizations, as well as electrophysiological signals such as pulse, electromyography, and electrocardiograms.^[^ ^7^ ^]^ Various mechanisms, including resistive, capacitive, piezoelectric, and tribo‐electric, have been explored for developing strain sensors.^[^ ^8^, ^9^ ^]^ Among these, resistive‐based flexible strain sensors are highly desirable due to their straightforward and facile preparation processes.^[^ ^10^ ^]^ Key parameters for evaluating the performance of a strain sensor include sensitivity and sensing range.^[^ ^11^ ^]^ Applications such as pulse detection require high sensitivity, while activities like joint motion monitoring, particularly in soft and industrial robots, demand high stretchability with uniform resistance change. Additionally, thermal properties, particularly thermal dissipation, are crucial for mitigating temperature influences on resistance and improving wear comfort.

To develop high‐performance strain sensors, numerous strategies have been explored. Initially, depositing nanoscale metal/semiconductor films on flexible substrates^[^ ^12^, ^13^, ^14^ ^]^ were investigated to construct deformable conductive paths within these sensors. However, the low stretchability of these conductive layers restricted their capability to withstand large strains. Consequently, recent research has shifted focus toward incorporating conductive fillers, such as metal nanowires,^[^ ^15^ ^]^ carbon nanotubes (CNTs),^[^ ^16^ ^]^ graphene,^[^ ^17^ ^]^ and carbon black,^[^ ^18^ ^]^ within highly stretchable polymer matrix, such as thermoplastic polyurethane^[^ ^19^ ^]^ and Ecoflex,^[^ ^20^ ^]^ forming deformable percolation networks. Based on this approach, numerous research works have been published on flexible resistive strain sensors, demonstrating either ultrahigh sensitivity,^[^ ^21^ ^]^ ultra‐fast response speed,^[^ ^22^ ^]^ large sensing range, or enhanced thermal properties.^[^ ^10^ ^]^ Nevertheless, challenges remain. For example, there is often a trade‐off between sensitivity and sensing range, as high sensitivity requires a sharp break within a fragile conductive network with a drastic change in resistance under small strain, while a wide sensing range demands a durable network that can deform continuously and uniformly across a wide strain range and maintain a stable gauge factor (*GF*). Besides, it is crucial to regulate the heat generated during operation,^[^ ^10^ ^]^ since excessive heat and elevated temperature can influence the resistance, potentially compromising the accuracy of strain detection. Unfortunately, few studies have discussed about the thermal properties of reported sensors.

Overall, there remains an urgent demand for strain sensors that simultaneously exhibit outstanding stretchability, high sensitivity, and good heat dissipation performance. To address the above‐mentioned challenges, careful selection of appropriate fillers is one of the most significant aspects. Among various conductive fillers, CNTs stand out due to their unique sp_2_‐bonded honeycomb lattice and 1D structure, resulting in superior mechanical, thermal, and electrical properties.^[^ ^23^ ^]^ These attributes render CNTs particularly attractive as fillers for strain sensors. Moreover, the high aspect ratio of CNTs enables the formation of a conductive percolation network at a low content, which is desirable for minimizing defects and mitigating the negative impacts on the overall stretchability of the composite. To ensure uniform and substantial resistance changes and address the trade‐off between sensing range and sensitivity, the introduction of liquid metal (LM) presents a promising solution. LM, with its ability to adjust shape in tandem with matrix deformation,^[^ ^24^ ^]^ may bridge the gaps between adjacent CNTs even under large strains, expanding the functional strain range without sacrificing sensitivity. Moreover, due to the high thermal conductivity of LM,^[^ ^25^ ^]^ the LM@CNTs dual‐material network holds the potential to improve the heat dissipation ability of the sensor.

Another promising strategy for improving sensors is the development of multi‐functionality. Apart from the conventional adherent type, where strain originates from the object to which the sensor is attached, strains can also be induced remotely by various external stimuli, including PH changes, electric fields, and magnetic fields. This multi‐functionality becomes especially attractive when integrated with appropriate shape design, which can be realized through additive manufacturing (AM) techniques. AM, particularly direct ink writing (DIW), has been widely applied in fabricating hydrogels and polymer nanocomposites,^[^ ^26^ ^]^ both of which are extensively explored in the synthesis of flexible strain sensors.^[^ ^27^, ^28^ ^]^ The combination of AM and intelligent materials, that can change shape, performance, or functionality in response to external stimuli, is the rapidly developing 4D printing technology.^[^ ^29^, ^30^, ^31^ ^]^ One of the simplest methods to create intelligent sensors is to incorporate magnetic iron powders into the resin. These powders, when attracted by an external magnetic field, will induce deformation in the sensor, resulting in a subsequent change in resistance and the generation of electric signals. With proper calibration, the sensor can be utilized to detect magnetic objects, making it inherently “multi‐functional”. In addition to 4D printing, another popular advancement of AM, namely multi‐material printing,^[^ ^32^ ^]^ can also be applied in sensor fabrication to expand its application scope. This can be achieved through a simple incorporation of a second functional material or part. For example, introducing a secondary material, such as an aluminum‐rich polymer‐based composite, in a multi‐path pattern during printing may enhance heat dissipation rates and improve the temperature stability of the sensor.

**Figure 1:**
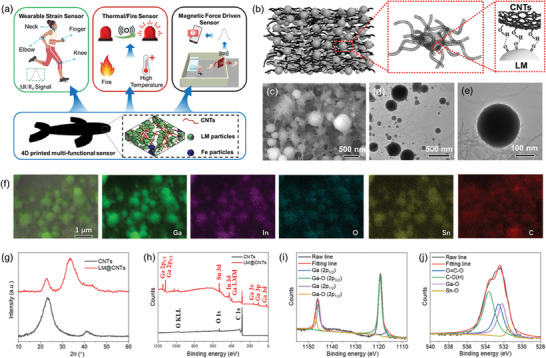
Characterizations of the LM@CNTs mixture. a) Multi‐functionality of 4D printed sensors. b) Schematic of the interaction between LM and CNTs. c) SEM and d) TEM image of the LM@CNTs mixture (LM: CNTs = 80: 1 by weight, the same as below). e) TEM image of a single LM particle. f) EDS mapping of the LM@CNTs mixture. g) XRD spectrum of CNTs and LM@CNTs. h) XPS spectrum and i) high‐resolution Ga 2p and j) O 1s.

In this work, a composite resin has been developed by blending CNTs, LM, and micron‐scale iron powders with Ecoflex 00‐10. Subsequently, the uncured resin was fabricated into desired shapes via DIW and then cured through a heating process. With proper control of composition, a unique balance of high sensitivity, stability, and multifunctionality can be achieved, unlocking the sensor's potential in multifunctional sensing devices. The incorporation of LM as a conductive bonding agent between CNTs creates a stable, continuous conductive network within the Ecoflex matrix. This configuration not only enhances electrical conductivity but also improves temperature sensitivity due to the high thermal conductivity of the LM. Additionally, the inclusion of iron particles introduces magnetic responsiveness, enabling multifunctional sensing capabilities—such as strain, temperature, and magnetic field detection—within a single, highly flexible structure. This combination of strain and magnetic sensing within a thermally stable elastomer offers versatility and adaptability across various applications (**Figure** **1**a). In summary, these findings are poised to pave the way for the development of the next generation of sensors, aligning well with the prevailing trend of function integration in smart electronic devices.

## 2 Results and Discussion

### 2.1 Material Characterizations

In order to find the optimal ratio for preparing the filler for wearable sensors, different amounts of CNTs were mixed with fixed‐weight LM. The resulting hybrid was named LM@CNTs‐n, where n denotes the mass (unit: 0.01 g) of CNTs in 2 g LM. A strong interaction between CNTs and LM is essential for the sensor to function as intended. CNTs and LM can interact with each other through the formation of hydrogen bonds between hydroxyls on CNTs and the oxide layer on LM particles,^[^ ^33^ ^]^ as depicted illustratively in Figure 1b. The SEM image is shown in Figure 1c, indicating a good dispersion of LM in the CNTs network, where LM particles are partially covered by CNTs. This was further verified by the TEM image (Figure 1d). The average size of LM particles is less than 1 µm, suggesting a high‐quality droplet breakup of LM during sample preparation. In the TEM image of a single LM particle (Figure 1e), a thin oxide layer with few attached CNTs is clearly visible on the surface. This attachment verifies the good compatibility between CNTs and LM. The existence of oxide layer was further verified by EDS maps (Figure 1f), where the mapping of O overlaps well with that of Ga, In, and Sn. Notably, the mapping of C also overlaps with that of metal elements, further suggesting that CNTs are adhered to the LM particle surface. Figure S1 (Supporting Information) shows that as the CNTs content in the hybrid increases, they progressively entangle and cover the LM particles. This phenomenon demonstrated that a certain content of LM can suppress the entanglement of CNTs. Moreover, the electrical conductivity of all fabricated LM@CNTs hybrids is excellent (Figure S2, Supporting Information).

The XRD spectrum of both CNTs and LM@CNTs (LM: CNTs = 80: 1 in weight) are presented in Figure 1g. Within the LM@CNTs spectrum, diffraction peaks corresponding to both LM and CNTs are discernible. The broad peak of LM indicates a strong amorphous behavior, consistent with findings from another study.^[^ ^34^ ^]^ However, metal oxide peaks are not clearly observed, likely due to the merging of these peaks within the wide amorphous peak. To explore the potential existence of metallic oxides and the interaction between CNTs and LM, the surface chemical composition was further analyzed using XPS (Figure 1h). In the LM@CNTs spectrum, the additional Ga peaks (Ga 2p_3/2_ (1119.9 eV), Ga 2p_1/2_ (1146.5 eV), Ga 3s (160.7 eV), Ga 3p (105.6 eV), Ga 3d (20.4 eV)^[^ ^35^, ^36^, ^37^ ^]^), Sn peaks (Sn 3d (531.5 eV)^[^ ^38^ ^]^), and In peaks (In 3d (445.0 eV)^[^ ^39^ ^]^) are observed apart from C 1s (284.8 eV) and O 1s (532.8 eV).^[^ ^21^ ^]^ The resolved peaks of Ga‐O within the Ga 2p spectrum (Figure 1i) confirm the presence of Ga_2_O_3_, aligning with the observation of oxide layer in Figure 1e. Furthermore, the O 1s (Figure 1j) spectrum displays four resolved peaks of O = C─O (532.6 eV), C─O(H) (533.8 eV), Ga─O (532.3 eV), and Sn─O (531.5 eV^[^ ^38^, ^40^, ^41^ ^]^), suggesting the existence of SnO_2_ apart from Ga_2_O_3_. Surprisingly, the XPS peak for In─O was not observed within the O 1s spectrum, potentially ascribed to the relatively low indium content near the upper surface of LM particles.

### 2.2 Mechanical Performance

**Figure 2:**
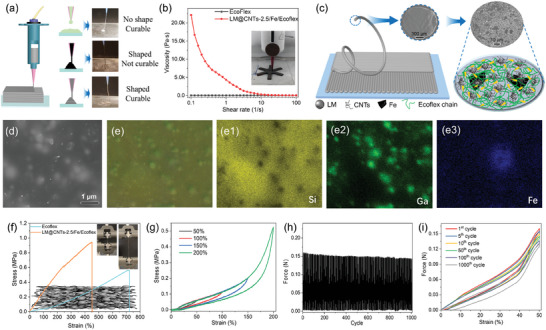
Basic properties of LM@CNTs/Fe/Ecoflex ink before and after curing. a) Shape ability and curability of resin in different compositions. b) The viscoelastic behavior of uncured Ecoflex and LM@CNTs/Fe/Ecoflex ink. c) Illustration of the structure of DIW extruded LM@CNTs/Fe/Ecoflex sample and filaments. d) SEM image and (e‐e3) EDS mapping of cryogenically ruptured surface of LM@CNTs/Fe/Ecoflex sample. f) Tensile curves for Ecoflex and LM@CNTs/Fe/Ecoflex sample. g) The 1st strain‐stress loop for LM@CNTs/Fe/Ecoflex under various strains from 50% to 200%. h) The long‐term force curve of the LM@CNTs‐2.5/Fe/Ecoflex sample with 1000 stretch and release cycles under a 50% strain. i) The force‐strain loop for LM@CNTs‐2.5/Fe/Ecoflex in 1st, 5th, 10th, 50th, 100th and 1000th stretch and release cycle.

In the context of DIW, the viscoelastic characteristics of inks are crucial factors that significantly impact both printability and the final quality of the printed product. An ideal DIW ink should demonstrate shear‐thinning behavior, wherein it maintains a high viscosity at rest to preserve its shape after extrusion, while exhibiting a notable reduction in viscosity under high shear rates to facilitate smooth extrusion.^[^ ^42^ ^]^ The rheological behavior of inks with different compositions is presented in Figure S3a (Supporting Information). The good flowability of pure Ecoflex resin enables easy extrusion from the syringe, solidifying within 10 mins at 80 °C. However, printed Ecoflex structures collapsed immediately, rendering it unsuitable for DIW (**Figure** **2**a). Conversely, Ecoflex composites containing CNTs maintain their original shape after extrusion, but curability becomes challenging, particularly when the CNTs content exceeds 1.5 wt.% (refer to the mass of Ecoflex resin, the same below). This phenomenon can be ascribed to the increased storage modulus due to the entangled CNTs, which hinders the molecular movement of the composites (Figure S3b, Supporting Information), obstructing the crosslinking between the two reactive components in Ecoflex. Consequently, resin with CNTs alone proved unsuitable for sensor fabrication in this work. The introduction of LM untangles the nanotubes, facilitating a smooth curing reaction within 3 h at 80 °C. Additionally, iron particles with an average diameter of 30–40 microns were added into the composite to impart magnetic‐responsive performance. Further characterization revealed that the introduction of iron particles in low content had a negligible impact on both printability and curability. Therefore, LM@CNTs/Fe/Ecoflex resin was adopted as the feedstock for DIW sensor fabrication, owing to its excellent printability and curability. Detailed comparisons between the printability, curability, and conductivity among different formulations can be found in Figure S4 (Supporting Information).

To identify the optimal formulation of LM@CNTs/Fe/Ecoflex, various samples in different compositions were prepared and evaluated with a rheometer (Figure 2b). As expected, an increased filler content elevated the viscosity of the composite resin, impairing printability. When the CNTs content exceeded 3 wt.% in the mixture, the ink became difficult to extrude, resulting in a failed fabrication. Notably, resins containing 1.5–2.5 wt.% CNTs exhibited smooth DIW printing. The extruded filament exhibited uniform thickness and texture, and accumulated layer by layer according to the pre‐designed shape to form the sensor precursor. In the enlarged SEM image, the LM@CNTs/Fe hybrids were well dispersed in the Ecoflex matrix, illustrated schematically in Figure 2c. The cryogenically fractured surface of a cured sample (Figure 2d) confirmed the effective embedding of LM, CNTs, and iron powders in Ecoflex, demonstrating excellent compatibility with the matrix. EDS maps (Figure 2e) further validate the uniform dispersion of LM and iron particles within the composite.

Tensile curves depicting the mechanical behavior of cured Ecoflex and LM@CNTs/Fe/Ecoflex are presented in Figure 2f. With the addition of fillers, the elongation at break decreases from over 700 to 450%. Nevertheless, this strain range for Ecoflex^[^ ^43^ ^]^ composites remains suitable for most human motion detection applications, as well as for scenarios demanding adaptability to more extreme strain conditions, such as wearable devices on flexible surfaces that undergo frequent and large deformations. This dual functionality creates opportunities for applications in fields like soft robotics,^[^ ^44^ ^]^ where sensors need to detect extensive bending or stretching while maintaining reliable sensitivity and consistency. Despite the reduction in maximum strain, the well‐dispersed LM@CNTs hybrids toughen the Ecoflex, thereby enhancing its strength and extending its service life. The ability of LM particles to dynamically adjust their shapes in response to external forces enables them to conform to the deformation of the matrix, ensuring the robustness of the conductive paths formed by CNTs. Figure 2g depicts the strain‐stress curve of as‐fabricated sensor at various strains (50–200%) under a fixed strain rate (120 mm·min^−1^) for the first loading‐unloading cycle. Substantial hysteresis is evident across all cycles. Similar hysteresis was also reported in other literature,^[^ ^45^, ^46^ ^]^ that can be attributed to the significant and permanent microstructural changes occurring during the initial loading cycle. However, the stable curve (Figure 2h) obtained from the anti‐fatigue performance test of the LM@CNTs‐2.5/Fe/Ecoflex, with a total of 1000 tensile loading‐unloading cycles at a strain of 50%, indicates that the equilibrium state is swiftly reached within the first few cycles. While some hysteresis persists due to the incompatible deformation between the CNT/LM network and the polymer matrix, minimal differences observed between the 1st, 5th, 10th, 100th, and 1000th mechanical cycles (Figure 2i) confirm the mechanical durability of the printed sensors, demonstrating that most deformations during stretching can be recovered, which is highly desirable for practical applications.

To verify the flexibility and conductivity of the printed LM@CNTs‐2.5/Fe/Ecoflex sensor, it was integrated into a circuit with an LED bulb as a qualitative resistance indicator. The luminosity of the LED bulb exhibited marginal variation upon curvature and torsion of the printed sample (Figure S5a, Supporting Information). In contrast, a noticeable reduction in brightness ensued upon stretching the sample, followed by an immediate restoration (<0.5 s) to its initial luminosity upon removal of the external force (Figure S5b, Supporting Information). This phenomenon demonstrates the sensor's ability to be flexibly adhered to different parts of the human body with negligible resistance change. Once properly adhered, the resistance of the sensor would change with motion, and the distinct change in luminosity during stretching highlights its high strain sensitivity. Overall, it is a promising candidate for wearable strain sensors. To quantitively evaluate the sensing performance, electromechanical performance of the LM@CNTs‐2.5/Fe/Ecoflex sensor was further characterized, as presented in the subsequent section.

### 2.3 Electromechanical Performance of the Strain Sensor

**Figure 3:**
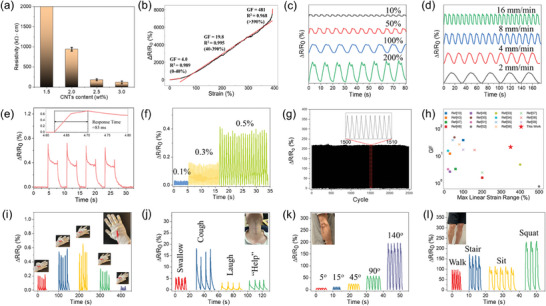
The sensing properties of LM@CNTs/Fe/Ecoflex sensor. a) Resistivity of sensors with different CNTs content. b) Relative resistance‐strain curve for LM@CNTs‐2.5/Fe/Ecoflex sensor. c) Relative resistance responses of the LM@CNTs‐2.5/Fe/Ecoflex sensor under cyclical stretch of various strains from 10% to 200% at a strain rate of 2 mm·min^−1^. d) Relative resistance responses of the LM@CNTs‐2.5/Fe/Ecoflex sensor under cyclical stretch of 100% at various strain rates from 2 to 16 mm·min^−1^. e) The response time for stretching. f) Relative resistance changes under cyclic stretch of tiny strains from 0.1% to 0.5%. g) The long‐term durability test of the LM@CNTs‐2.5/Fe/Ecoflex sensor with 2500 stretch and release cycles under a 50% strain. The insert plot shows the details of the 1500–1510 cycles. h) Comparison of the *GF* in the widest linear strain range with 15 recently reported strain sensors. i) The response in different gestures and j) in different throat motions. k) The response of elbow bending in different angles. l) The response of the knee in different actions.

The device performance is significantly influenced by the loading of fillers, particularly that of CNTs. As shown in **Figure** **3**a, an increase in CNTs content from 1.5 to 2.5 wt.% results in a rapid decrease in sample resistivity, dropping from over 2000 to 180 kΩ·cm. Further addition of CNTs from 2.5 to 3 wt.% results in only a marginal reduction in resistivity. Consequently, the percolation threshold for CNTs in this specific system is identified to be between 2 and 2.5 wt.%. For an optimal strain sensor, the filler content must be sufficiently high to form the percolation network, while remaining low enough to ensure desirable sensitivity and detection limits. Therefore, 2.5 wt.% is chosen for further investigation and characterization.

As mentioned above, two critical parameters frequently employed to evaluate strain sensor performance are sensitivity and maximum functional stretchability. Sensitivity is typically quantified by the *GF*, defined as *GF* = Δ*R*/(*R*·*ε*), where *ε* is the applied strain, Δ*R* is the resistance change corresponding to the strain, and *R* is the initial sample resistance without external disturbance. In this work, the maximum functional stretchability excludes the region where the *GF* is extremely large, a condition that occurs when the percolation network within the sample breaks completely, rendering the performance unreliable. A wide strain range with a constant and large *GF* is particularly desirable for practical strain sensors.

The Δ*R*/*R*‐*ε* curve of LM@CNTs‐2.5/Fe/Ecoflex (Figure 3b) exhibited two distinct linear regions. Under relatively small strains (0–40%), the CNTs percolation network deforms without breaking, resulting in a relatively low *GF* of 4.0. When the strain further increases (40–390%), the network begins to break, leading to a sharp increase in resistance and a substantial *GF* of 19.8. Beyond 390% strain, the *GF* reaches 481, rendering the sensor unrecoverable and unreliable. Therefore, the maximum functional stretchability was determined to be 390%. While the first linear region encompasses the strain range typical of most human activities,^[^ ^47^ ^]^ the second linear region extends over a wide range of 350% strain with high linearity (R^2^ = 0.995), making it highly desirable for applications such as motion sensing for soft robotics. Moreover, the high linearity within these two regions enhances detection precision, accommodating a certain degree of nonuniformity in strain due to friction or other restrictive forces. In comparison, at lower CNTs content (LM@CNTs‐1.5/Fe/Ecoflex and LM@CNTs‐2.0/Fe/Ecoflex), a significant increase in resistance is observed ≈100% strain, followed by pronounced signal scattering as the strain continues to increase (Figure S6a,b, Supporting Information). This behavior is attributed to the poor formation of the conductive network in these compositions, leading to complete network breakage at an early stage and resulting in extremely high resistance, close to the detection limit of the machine. This sharp increase in resistance, combined with the lack of strong linearity, renders these two compositions unsuitable for reliable strain sensing beyond 100%.

Figure 3c presents the electrical response of the sensor during a cyclic stretching‐releasing process. The peak height increases with increasing strain and remains stable even under 200% cyclic strain, indicating the high structural stability of the sensor. Furthermore, the electrical responses during cyclic processes with four different strain rates, i.e., 2, 4, 8, and 16 mm·min^−1^, were investigated. These regular and stable peaks in Figure 3d indicate that the resistance recovered as quickly as the loading cycle, further suggesting its excellent structural stability and high response speed. To go one step further, the sensor was tested under 1% strain at a high strain rate (500 mm·min^−1^) to evaluate its response time. As depicted in Figure 3e, the response time of the sensor is 83 ms, shorter than many recently reported studies.^[^ ^48^, ^49^, ^50^ ^]^ This rapid response can probably be attributed to the simple network deformation under small strain, eliminating the need for extra time to rebuild the percolation network.

The detection limit is another significant factor for detecting tiny yet essential physiological activities, such as heart beats and breathes. To evaluate the detection limit of the sensor, the relative resistance change was characterized in cyclic stretching‐releasing processes under three ultra‐low strains: 0.1%, 0.3%, and 0.5%. Surprisingly, even with a tiny strain of 0.1%, clear and stable peaks are observed, indicating an exceptionally low detection limit, as shown in Figure 3f. This competitive detection limit can be ascribed to the good structural integrity of the sample, which minimizes noise during operation. The low detection limit, coupled with the wide sensing range and high linearity, demonstrates the potential of this sensor in the detection of various human motions and physiological activities.

Durability and long‐term stability are essential considerations for industrial applications. Figure 3g shows the results of the cyclic stability test conducted on the sensor from 0% to 50% strain at 100 mm·min^−1^ strain rate. The relative resistance change stabilized rapidly after several initial cycles, to reach the equilibrium between the breakage and reconstruction of CNTs percolation networks. The stabilized relative resistance change is consistent with that shown in Figure 3b, confirming the superior reliability of the sensor. Moreover, the performance of the sensor remains stable even after 2500 cycles, indicating excellent long‐term reliability and stability.

Figure 3h presents a comparison of this sensor with fifteen recently reported resistive‐based sensors in terms of *GF* and maximum linear strain range. Compared to other sensors reported in literatures,^[^ ^10^, ^46^, ^51^, ^52^, ^53^, ^54^, ^55^, ^56^, ^57^, ^58^, ^59^, ^60^, ^61^, ^62^, ^63^ ^]^ the present sensor offers both a wide linear workable range (350%) and a high *GF* (19.8). Additionally, this sensor exhibits a short response time (83 ms) and a low detection limit. More details of this comparison are provided in **Table** **1**. Overall, this sensor demonstrates excellent comprehensive performance and emerges as a promising candidate for various motion detection applications.

**Table 1:** Detailed comparison of the sensor in this work with 15 recently reported sensors.

Materials	Stretchability [%]	Max. Linear Range [%]	Corresponding *GF*	Ref.
GNR/BNNS/TPU	330	60–160	35.7	10
CNTs/Ecoflex	20	0–20	14.5	46
CNTs/Ti_3_C_2_T_x_ MXene/PDMS	60.3	30–60.3	11.4	51
CNTs/PDMS	100	40–100	3.1	52
Organohydrogel (PAC‐Zn)	488	200–400	1.486	53
Graphene/cotton/PDMS	70	0–30	2.49	54
CNTs/GR/PDMS	120	3–57	45.6	55
Polypyrrole/β‐FeOOH/nylon	350	0–20	3.06	56
PPy‐silk fibroin/tannic acid	500	0–500	0.705	57
PAM‐3‐ TSASN‐LiCl hydrogel	1200	800–1200	4.5	58
CNTs/PDMS	45	0–45	35.75	59
Weave cotton fabric/Ecoflex	140	0–80	25	60
MXene /PVA‐CA‐based hydrogels	400	0–200	2.3	61
LM@MXene/UPAM	919	240–400	15.47	62
ANF‐PVA/APP	140	0–100	16	63
LM@CNTs‐2.5/Fe/Ecoflex	390	40–390	19.8	This work

Based on all the aforementioned properties, the practical application of the proposed sensor in monitoring various human motions is further investigated as a proof of concept. The real‐time output signal is stable and matches well with the movement of the finger (Video S1, Supporting Information), indicating that the as‐fabricated samples can effectively detect joint motions. In Figure 3i, five sensors are attached to five fingers of a glove, connected in parallel for signal collection. As each finger bends ≈90°, varying peak heights are observed, making this sensor‐equipped glove suitable for remote control, where distinct gestures can command a robot or a machine. Furthermore, if the signal of each finger is recorded individually, the sensor can also be utilized for gesture recognition or rehabilitation. Apart from finger motions, the sensor was also adhered to the throat for vocal cord motion detection. As depicted in Figure 3j, different motions, including swallowing, coughing, and laughing, generate rapid and distinguishable responses with different peak shapes and heights, facilitating easy recognition. In addition, the sensor was also fixed on elbows (Figure 3k) and knees (Figure 3l), and discernible responses corresponding to different motions were observed.

### 2.4 Thermal Performance of the Sensor

**Figure 4:**
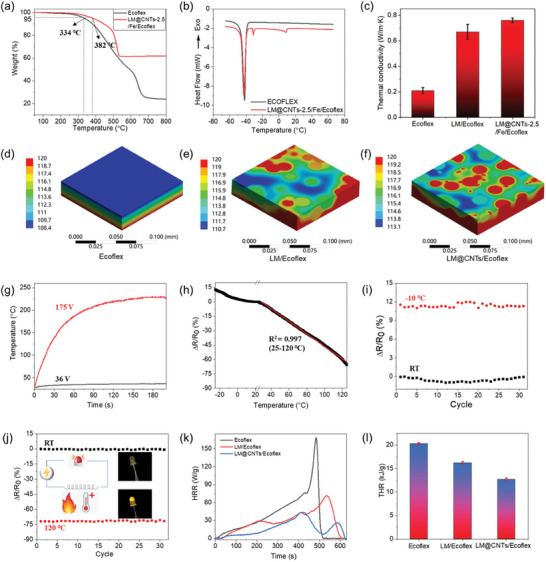
The thermal properties of LM@CNTs‐2.5/Fe/Ecoflex sensor. a) TGA curves of Ecoflex and LM@CNTs‐2.5/Fe/Ecoflex from 50 to 800 °C. b) DSC curves of Ecoflex and LM@CNTs‐2.5/Fe/Ecoflex from −70 to 70 °C. c) The thermal conductivity for Ecoflex, LM/Ecoflex and LM@CNTs‐2.5/Fe/Ecoflex. d) Simulated thermal equilibrium in thin (106 × 106 × 21 µm) sheets of Ecoflex, e) LM/Ecoflex, and f) LM@CNTs/Ecoflex on a heating stage at 120 °C. g) Temperature‐time curves for LM@CNTs‐2.5/Fe/Ecoflex under 36 and 175 V voltage. h) The relative resistance‐temperature curve for LM@CNTs‐2.5/Fe/Ecoflex. i) The thermal durability test of the LM@CNTs‐2.5/Fe/Ecoflex with 30 cycles between −20 °C to room temperature (RT). j) The thermal durability test of the LM@CNTs‐2.5/Fe/Ecoflex with 30 cycles between RT to 120 °C. k) *HRR* curves for Ecoflex, LM@Ecoflex, and LM@CNTs‐2.5/Fe/Ecoflex. l) Comparison of *THR* for Ecoflex, LM@Ecoflex, and LM@CNTs‐2.5/Fe/Ecoflex.

In addition to its outstanding performance as a strain sensor, LM@CNTs‐2.5/Fe/Ecoflex also exhibits superior thermal properties. TGA curves (**Figure** **4**a) indicate that the decomposition temperature (corresponding to 5% weight loss) increases from 334 to 382 °C (343 °C for LM/Ecoflex, Figure S7a, Supporting Information) due to the addition of thermally stable CNTs, LM and Fe powders. This enhanced thermostability ensures the safe application of the material in higher‐temperature environments. The DSC curve (Figure 4b) of Ecoflex showed a strong endothermic peak at ≈−42 °C, corresponding to the glass transition temperature (*T*_g_).^[^ ^64^ ^]^ The addition of LM@CNTs/Fe introduces two extra peaks but has trivial impact on *T*_g_. The additional peak in the DSC curve of LM@CNTs‐2.5/Fe/Ecoflex at 8.0 °C can be attributed to the melting of LM. The broadening of this peak results from the small particle size of LM and the interaction between LM and CNTs, which shifts the melting point of LM. Another unexpected peak at −31.2 °C, which also appeared within the DSC curve of LM/Ecoflex sample (Figure S7b, Supporting Information), probably originates from the impurities within the LM, such as metallic oxides.

A temperature elevation during strain sensor operation, caused by internal friction and electric heating, has been reported to be non‐negligible.^[^ ^10^ ^]^ Thus, effective thermal dissipation is crucial for both sensing accuracy and wearing comfort. In this work, the thermal conductivity of Ecoflex, LM/Ecoflex, and LM@CNTs‐2.5/Fe/Ecoflex were measured to investigate the influence of LM and CNTs on the sample's thermal dissipation capability. The results are presented in Figure 4c. The inclusion of LM remarkably enhanced the thermal conductivity of Ecoflex, and this value further increased with the additional inclusion of CNTs. This enhancement can be attributed to the excellent conductivity of LM and CNTs, as well as the formation of LM@CNTs dual‐material network, which provides an interconnected path for better heat conduction and release.

The effects of LM and CNTs were further verified through simulations (Figure 4d–f) of the steady thermal state in three thin sheets made of pure Ecoflex, LM/Ecoflex, and LM@CNTs/Ecoflex. The bottom surface of all sheets was maintained at a constant temperature of 120 °C (Other details of the simulation are listed in Table S1, Supporting Information). The presence of a red high‐temperature region within the upper surface of LM/Ecoflex sheet indicates a significant improvement in thermal conduction due to the introduction of LM particles into the Ecoflex matrix. The further expansion of red region in LM@CNTs/Ecoflex can be attributed to the formation of LM@CNTs network, facilitated by the high aspect ratio of CNTs. To validate the simulation results, samples with identical shapes (30 × 20 × 2 mm) but different compositions were placed on a heating stage set at 120 °C, and the temperature change was recorded (Figure S8, Supporting Information). The result suggests that despite similar heating and cooling rate at the start and end of the three curves, the three materials stabilized at different temperatures, with 90 °C for Ecoflex, 95 °C for LM/Ecoflex, and 99 °C for LM@CNTs‐2.5/Fe/Ecoflex. This sequence is consistent with the simulated average temperature of the upper surface of the above‐mentioned three thin sheets (Figure S9, Supporting Information). Considering the comparable heat dissipation of all three samples that is dominated by the air convection at the Ecoflex surface, the equilibrium surface temperature would be predominantly governed by the thermal conductivity of the material. Consequently, materials with higher thermal conductivity can support elevated surface temperatures.

To evaluate the thermal performance of the as‐fabricated LM@CNTs‐2.5/Fe/Ecoflex sensor during practical operation, electrothermal tests were conducted at two different voltages (Figure 4g). Under 36 V, the temperature increase was minimal, demonstrating the sensor's excellent thermal dissipation capability. When 175 V is applied, the temperature rises significantly due to the square relationship between the electric heat and voltage, stabilizing at 230 °C without any visible damage. This high‐temperature resistance underscores the sensor's potential to perform at elevated temperatures.

For resistive strain sensors operating across varying temperatures, it is essential to determine the effect of temperature on resistance beforehand to enable accurate corrections, especially for materials whose resistance is highly sensitive to temperature fluctuations. The resistance, normalized with the value at room temperature (RT, 23 °C), is presented as a function of temperature in Figure 4h. A high linearity with an R^2^ of 0.997 is observed within a wide temperature range from 20 to 120 °C, with a coefficient of −0.65%/°C, implying a decrease in resistance with increasing temperature. This resistance drop can be attributed to the enhancement of charge carrier mobility within the CNTs network at elevated temperatures. As the temperature rises, the electrical resistance of CNTs decreases significantly, resulting in measurable variations in the sensor's overall resistance. For a wearable strain sensor designed for human motion detection, a temperature variation from 20 to 40 °C would result in a relative resistance change of 13%, corresponding to 3.2% strain in the first linear region or 0.65% in the second one. These substantial strain deviations indicate the necessity of separating thermal and strain contributions to achieve a high sensing accuracy. Fortunately, this linearity facilitates the straightforward decoupling of thermal and mechanical signals. To compensate for temperature variations, an unstretched reference sensor (e.g., mounted on a rigid plate) can be added. Additionally, given the distinct patterns of thermal‐ and strain‐induced signals, advanced signal processing methods, such as machine learning algorithms,^[^ ^65^ ^]^ can be employed. These algorithms can recognize and isolate patterns specific to temperature or strain changes, analyzing data based on response rates and magnitudes under varying conditions, thereby effectively decoupling the two signals.

To go one step further, the stability and repeatability of LM@CNTs‐2.5/Fe/Ecoflex sensor were characterized under heating‐cooling cycles. The result is shown in Figure 4i,j. Across 30 cycles in both low (−20 °C to RT) and high (RT to 120 °C) temperature regions, the relative resistance change remained consistent. This stable performance, coupled with the wide linear range up to 120 °C, suggests that this sensor could also be used as a promising temperature sensor. For example, it could be integrated into a fire alarm system, where an appropriate current threshold can be pre‐determined based on specific requirements. A signal would be generated if the current surpasses the threshold, indicating a rising temperature and the potential occurrence of a fire (Figure 4j). Video S2 (Supporting Information) shows a noticeable change in brightness when the printed samples were subjected to elevated temperatures.

Another crucial characteristic of a thermal sensor is its flammability, typically assessed by the heat release rate (*HRR*) curve.^[^ ^66^ ^]^ It is essential to avoid a large and wide heat release rate peak to mitigate fire growth. The *HRR* curves for Ecoflex, LM/Ecoflex, and LM@CNTs‐2.5/Fe/Ecoflex are presented in Figure 4k to assess the influence of LM on flammability, since unmodified CNTs are known to be highly flammable.^[^ ^67^ ^]^ The *HRR* curve for Ecoflex exhibits a gradual increase over time, reaching a peak at ≈480 s. This sharp heat release peak is undesirable for a thermal sensor, especially in the context of fire risks. The addition of LM greatly reduced the strength of the highest *HRR* peak and lowered the total heat release (*THR*). Moreover, the appearance of the *HRR* peak was delayed after the incorporation of LM, which helps mitigate flame growth. Interestingly, when the CNTs were introduced, the *THR* further decreased, despite the appearance of an additional peak. Besides, the CNTs significantly slowed the heat release rate during the first 200 s, avoiding rapid flame spread in the early stage. The comparison of THR for different compositions was summarized in Figure 4l. Overall, the anti‐flammability of the current sample was enhanced remarkably as compared to that of pure Ecoflex.

### 2.5 Magnetic Performance of the Sensor

**Figure 5:**
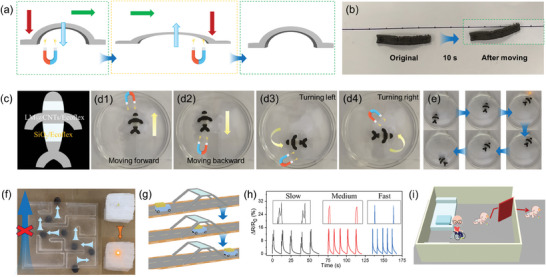
Magnetic performance of LM@CNTs‐2.5/Fe/Ecoflex sensor. a) The “crawling caterpillar” scenario, b) where a specifically designed sample is guided by magnetic field to move like a caterpillar. c) The “swimming fish” scenario, where a multi‐material printed fish carrying a wire is guided by magnetic field to “swim” (d1) forward, (d2) backward, (d3) left, and (d4) right in the water and (e) form a circuit. f) The “ball pass maze” scenario, where a sample ball carrying a wire is guided by magnetic field through a maze to form a circuit. g) The schematic scenario for magnetic‐based location and speed detection of cars. h) Electrical responses for cars in different speeds. i) The schematic application for home monitoring of vulnerable elders and children.

Due to the addition of iron particles, this material exhibits magnetic responsiveness and can be easily manipulated by magnets. Three tests were performed for verification, including “crawling caterpillar” (**Figure** **5**a,b), “swimming fish” (Figure 5c–e), and “ball pass maze” (Figure 5f). In the first scenario, a simple magnetic‐driven motion was performed to mimic the movement of caterpillars. The caterpillar‐like sample was designed and fabricated via DIW. Under the synergistic effect of the friction and magnetic field, the sample moved forward through a cyclic shift between crouching and stretching (Video S3, Supporting Information). Notably, the dual‐color “fish” (Figure 5c) in the second trial was fabricated by “multi‐material printing”. The dark part is LM@CNTs‐2.5/Fe/Ecoflex while the white part is SiO_2_‐loaded Ecoflex. A good compatibility was achieved between the two components due to the same matrix used for both materials. This trial highlights the advantages of AM for strain sensor fabrication, enabling the integration of multiple functionalities and the creation of complex geometries by combining different functional materials.

The fabricated multi‐material aquatic entity exhibits dynamic aquatic locomotion capabilities under the influence of remote external magnetic fields, executing intricate maneuvers including lateral movements (i.e., left and right turns), forward propulsion, and retreat (Figure 5d and Video S4, Supporting Information). To validate its operational efficacy, the printed fish incorporates embedded wiring infrastructure. Guided by remote manipulation via external magnetic fields, the entity accurately navigates to predetermined locations, establishes electrical connectivity, thereby illuminating the integrated LED bulb. Upon completion of the conductive path connection task, the multi‐material aquatic entity autonomously returns to its initial position, propelled by the exertion of external magnetic forces (Figure 5e and Video S5, Supporting Information). This demonstration underscores the synergistic potential of integrating multi‐material AM, conductive elements, and magnetic field propulsion, offering promising avenues for practical applications in real‐world scenarios. Furthermore, a demonstration involving the navigation of a printed sphere through a maze (Figure 5f and Video S6, Supporting Information) illustrates the potential for precise manipulation of printed components via magnetic influence, showcasing diverse application prospects for the entire system.

While simple magnetism alone may not distinguish this material from other flexible magnetic materials, its integration with strain sensing introduces versatility, enabling applications in location and speed detection. As shown in Figure 5g and Video S7 (Supporting Information), a sensor was fixed at a gate. When a toy car with a magnet attached at the top passed through the gate, an electric signal was generated due to the attraction between the magnet and sensor, resulting in strain and resistance change. Notably, different peak shapes were observed for three different speeds, qualitatively labeled slow, medium, and fast (Figure 5h). For slow speed, three sub‐peaks appeared within a single signal, corresponding to attraction, drag, and release stage, respectively. As speed increased, only one broad peak was clearly visible, attributed to the merging of peaks due to the higher speed. Further increasing the speed led to sharper peaks, indicating a more transient interaction between the sensor and moving objects. This result suggests that, due to the introduction of iron particles, the sensor can be used for semi‐quantitative location and speed detection based on the number and width of peaks within a signal. This characteristic could be valuable for home care, allowing remote monitoring of the location of vulnerable children or elders to prevent unwanted accidents, as schematically depicted in Figure 5i.

In addition to serving as a magnetic field sensor, this capability can work synergistically with the strain and thermal sensing functions, extending the sensor's applicability to more sophisticated scenarios. For example, in a rehabilitation setting, a wearable device equipped with these sensors could simultaneously track joint motion, skin temperature, and magnetic field variations from external equipment to monitor proximity or alignment during exercises, providing a comprehensive assessment of the user's health. In soft robotics, robots operating in dynamic environments could benefit from multi‐functional feedback. For instance, strain sensing can monitor the robot's flexibility, temperature sensing can detect excessive heat during prolonged use or in high‐temperature environments, and magnetic field detection can support navigation and orientation, especially in confined spaces. Additionally, industrial applications also benefit from this versatility, as equipment monitoring often involves high temperatures and mechanical stress. The presented sensor's ability to provide simultaneous strain, temperature, and magnetic feedback is valuable for ensuring operational safety and the early identification of performance issues. The multi‐functionality of this sensor offers numerous advantages over the recently reported sensor with similar material system.^[^ ^68^ ^]^

## 3 Conclusion

In summary, this work presents a flexible strain sensor LM@CNTs‐2.5/Fe/Ecoflex by incorporating CNTs, LM, and iron powders into the Ecoflex matrix. By carefully controlling the composition, an uncured resin with a strong shear‐thinning behavior which is suitable for DIW, and a cured sensor with excellent electromechanical, thermal, and magnetic performance were fabricated. A wide linear strain range of 350% with a stable and high *GF* of 19.8 was achieved, together with an ultralow detection limit of 0.1% strain and a low response time of 83 ms. Besides, this material exhibits attractive thermal performance, showcasing high‐temperature resistance (>300 °C), improved thermal conductivity, and a linear resistance‐temperature relationship. These characteristics make the LM@CNTs‐2.5/Fe/Ecoflex sensor a promising candidate for high‐temperature applications. Finally, the addition of iron particles imparts strong magnetic responsiveness to the material. The synergistic combination of magnetism and strain sensing makes the sensor suitable for location and speed detection, suggesting potential applications in home care. Moreover, with the aid of DIW, sensors with complex shapes and multiple functional materials can be fabricated, significantly expanding the sensor's functionality and potential applications. Overall, the sensor's dynamically adaptive response to temperature and magnetic fields ensures reliable performance across diverse environmental conditions and applications, demonstrating the superiority of 4D printing. Thus, we foresee that the combination of AM techniques and multi‐functional materials is envisioned to greatly improve the performance of next‐generation sensors.

## 4 Experimental Section

### Materials

MWCNTs were purchased from Chengdu Organic Chemicals Co. Ltd. (China). Ecoflex 00‐10 was purchased from Smooth‐On Inc (USA). LM (GaInSn alloy) was purchased from Dongguan Dingguan Metal Technology Co., Ltd (China). Iron powder (1000 mesh) was purchased from Nangong Daguang Welding Material Co., Ltd. (China). Large particles of iron powder were removed with a 200‐mesh sieve prior to use. All other materials were used as received.

### Preparation Methods

The preparation of LM@CNTs‐2.5/Fe/Ecoflex ink began with the dispersion of LM and CNTs. LM and CNTs were introduced into a sufficient amount of hexane, which was then ultrasonically dispersed with a sonicator (Q500‐220, Qsonica, USA) for 30 min. After ultrasonication, the supernatant was carefully removed. Subsequently, iron powders and Ecoflex 00‐10 part A were added into the remaining solution. The obtained mixture was stirred at 1000 rpm at 80 °C for hexane evaporation until it formed a slurry. The Ecoflex 00‐10 part B was then added into the mixture and stirred with a glass rod. Finally, the resulting mixture underwent degassing for 1 h using a rotary vane vacuum pump (Edwards RV12, Edwards Vacuum, UK) to completely remove the remaining hexane. The prepared ink was stored below 18 °C to prevent curing. The weight ratio of CNTs: LM: Iron powder: Ecoflex 00‐10 part A: Ecoflex 00‐10 part B was 1: 80: 6: 20: 20.

The prepared LM@CNTs‐2.5/Fe/Ecoflex ink was then fabricated into desired shapes using a direct ink printer (DIW) (BIO X 3D Bioprinter, Cellink AB, Sweden), and a tapered nozzle (20G) with an open diameter of 0.60 mm was adopted. A constant printhead speed of 5 mm s^−1^ was employed for all inks, while a pressure of 700, 500, 350, and 250 kPa was adopted for inks containing 3.0, 2.5, 2.0, and 1.5 wt.% CNTs, respectively. The shaped precursors were cured in an oven at 80 °C for 3 h to obtain the final sensors.

### Characterizations of LM@CNTs/Fe/Ecoflex Ink

The morphology and microstructure of dispersed CNTs and LM, as well as the LM@CNTs/Fe/Ecoflex ink were observed using a scanning electron microscope (SEM) (JSM‐5600 LV, JEOL, Japan). The morphology of LM@CNTs was further observed using a transmission electron microscope (TEM) (2010 HR & UHR, JEOL, Japan). The crystal structure of CNTs and LM@CNTs was characterized with an X‐ray diffractometer (XRD) (D8 Advance, Bruker, USA). The surface chemical elements and corresponding chemical states of CNTs and LM@CNTs were characterized with an X‐ray photoelectron spectroscope (XPS) (AXIS Supra, Kratos Analytical, USA). The viscoelastic behavior of the ink was measured with a shear rate ramp from 0.1 to 100 s^−1^ by a rotational rheometer (DHR‐2, TA Instruments, USA) with a parallel plate geometry of 40 mm in diameter.

### Characterizations of LM@CNTs/Fe/Ecoflex Sensors

The microstructure of cytogenic fractured surface of LM@CNTs/Fe/Ecoflex sensors was characterized by SEM (JSM‐5600 LV, JEOL, Japan). The four‐probe conductivity meter (HPS2662, HELPASS, China) with a measuring range from 0 to 2000 kΩ·cm was used to test the conductivity of printed sensors. The resistance of sensors was measured with a source meter (Keithley 2450, Tektronix, USA) with a source voltage of 15 V. The mechanical properties of printed sensors were tested with a universal testing machine (Instron 3366, Instron, USA). Thermogravimetric analysis (TGA) was conducted with a thermogravimetric analyzer (Q500, TA Instruments, USA), using samples ≈10 mg (obtained by cutting from DIW‐printed sensors), which were heated from 50 to 800 °C at 20 °C min^−1^ under nitrogen conditions. Differential scanning calorimetry (DSC) was performed with a differential scanning calorimeter (Q200, TA Instruments, USA), using samples ≈10 mg (obtained by cutting from DIW‐printed sensors), which were heated from −70 to 150 °C at 10 °C min^−1^ under nitrogen conditions. The temperature of sensors during heating and cooling was recorded with an infrared camera (A655sc, FLIR systems, USA). The thermal conductivities of samples were tested by a hot‐disk thermal analyzer (TC 3000E, Xia Xi Technology, China) at 25 °C, adopting the transient plane source technique. The micro‐scale combustion test was conducted on GOVMARK MCC‐2 following the ASTM D 7309‐07 standard to test samples of 20 mg. The same rheometer and parallel plate setup were used to measure the storage and loss modulus of the ink. A shear stress ramp from 0.1 to 10 000 Pa was applied at a constant frequency of 1 Hz.

## Conflict of Interest

The authors declare no conflict of interest.

## Supporting information



Supporting Information

Supplemental Video 1

Supplemental Video 2

Supplemental Video 3

Supplemental Video 4

Supplemental Video 5

Supplemental Video 6

Supplemental Video 7

## Data Availability

The data that support the findings of this study are available from the corresponding author upon reasonable request.
