# Supplementary material for: Ultraflexible Sensor Development via 4D Printing: Enhanced Sensitivity to Strain, Temperature, and Magnetic Fields
Source: Adv Sci (Weinh). 2024 Dec 24;12(7):2411584. doi: 10.1002/advs.202411584 (PMC11831529; doi:10.1002/advs.202411584)
Supplement: Supplementary file 1 — Supporting Information [file ADVS-12-2411584-s004.docx]

**Ultraflexible Sensor Development via 4D Printing: Enhanced Sensitivity to Strain, Temperature, and Magnetic Fields**

*Yanbei Hou, Hancen Zhang, and Kun Zhou**

Y. Hou, K. Zhou

Singapore Centre for 3D Printing

School of Mechanical and Aerospace Engineering

Nanyang Technological University

Singapore 639798, Singapore

Email: [kzhou@ntu.edu.sg](mailto:kzhou@ntu.edu.sg)

H. Zhang

Environmental Process Modeling Centre

Nanyang Environment and Water Research Institute

Nanyang Technological University

Singapore 639798, Singapore


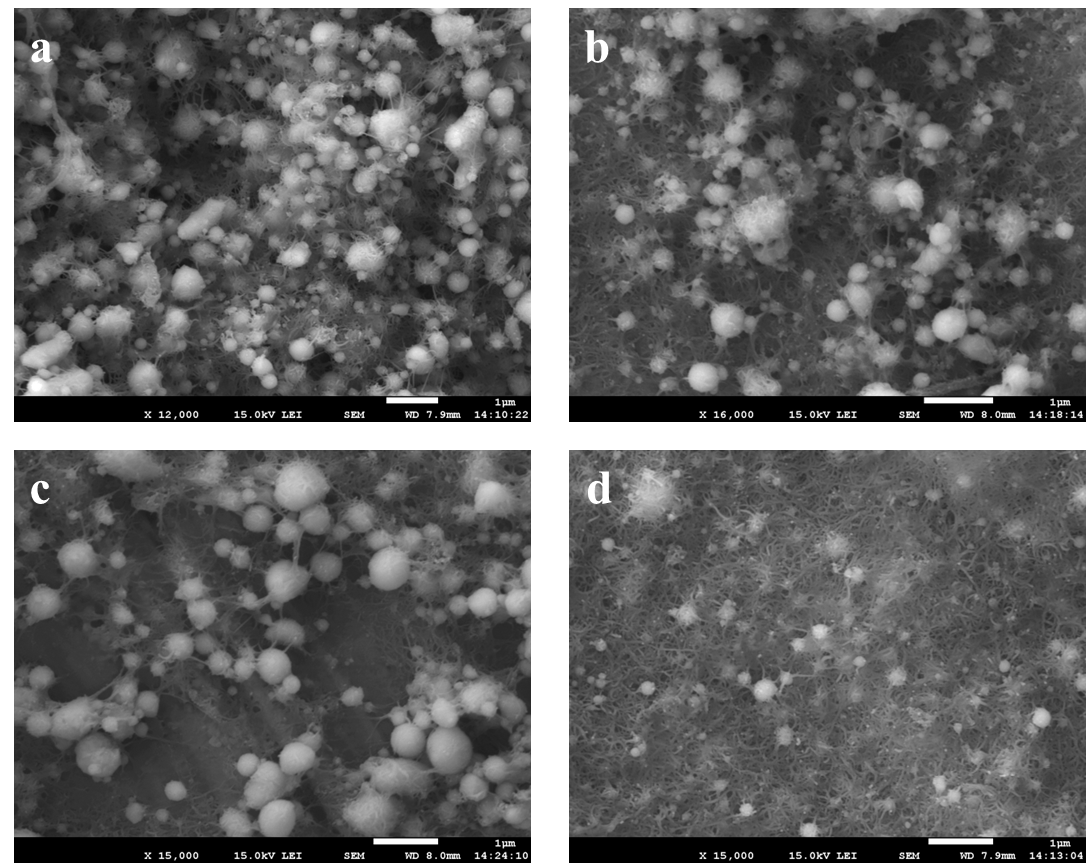


**Figure S1.** The SEM image for (a) LM@CNTs-1.5, (b) LM@CNTs-2.0, (c) LM@CNTs-2.5 and (d) LM@CNTs-3.0.


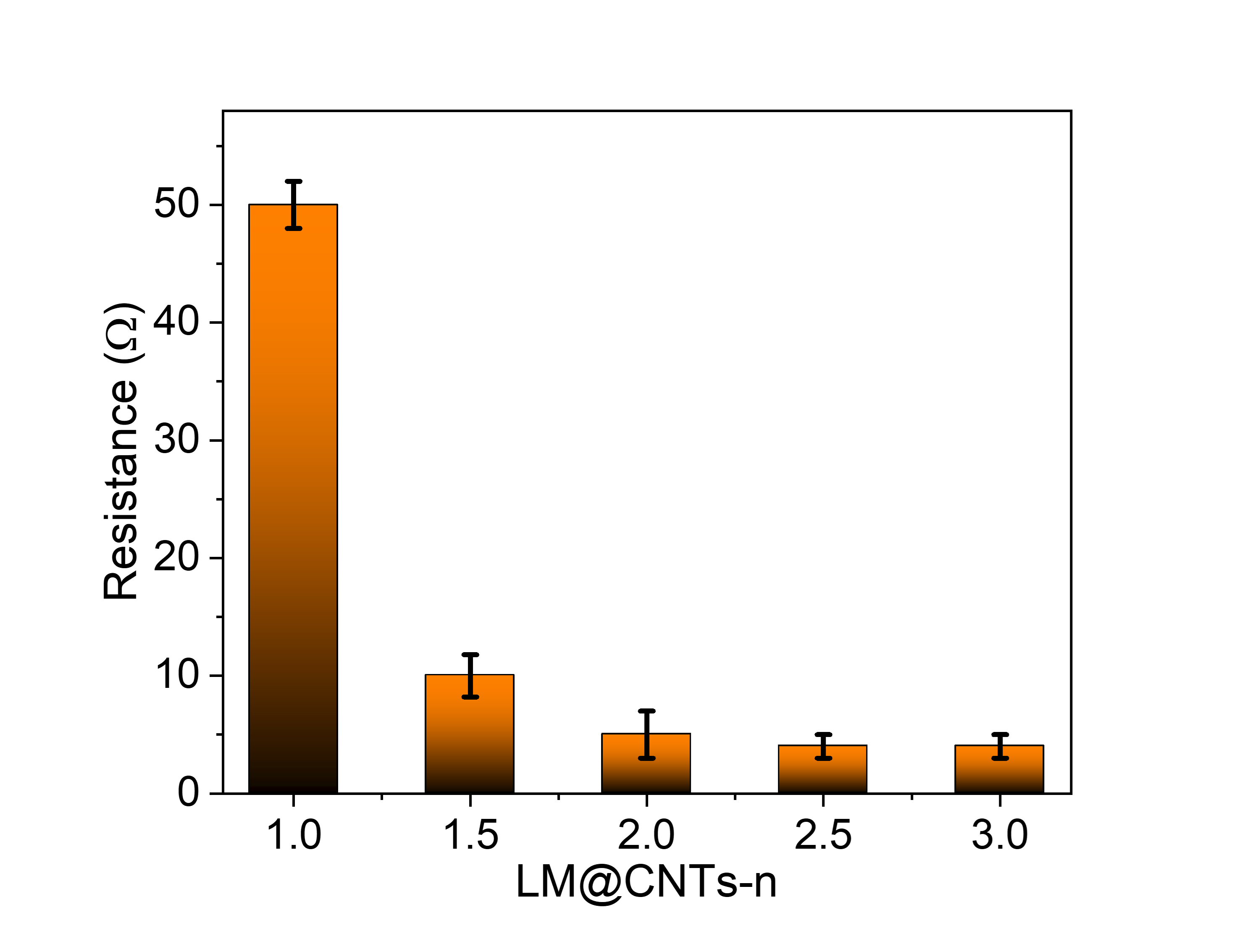


**Figure S2.** Electrical resistance for LM@CNTs-n (n from 1.0 to 3.0).

**
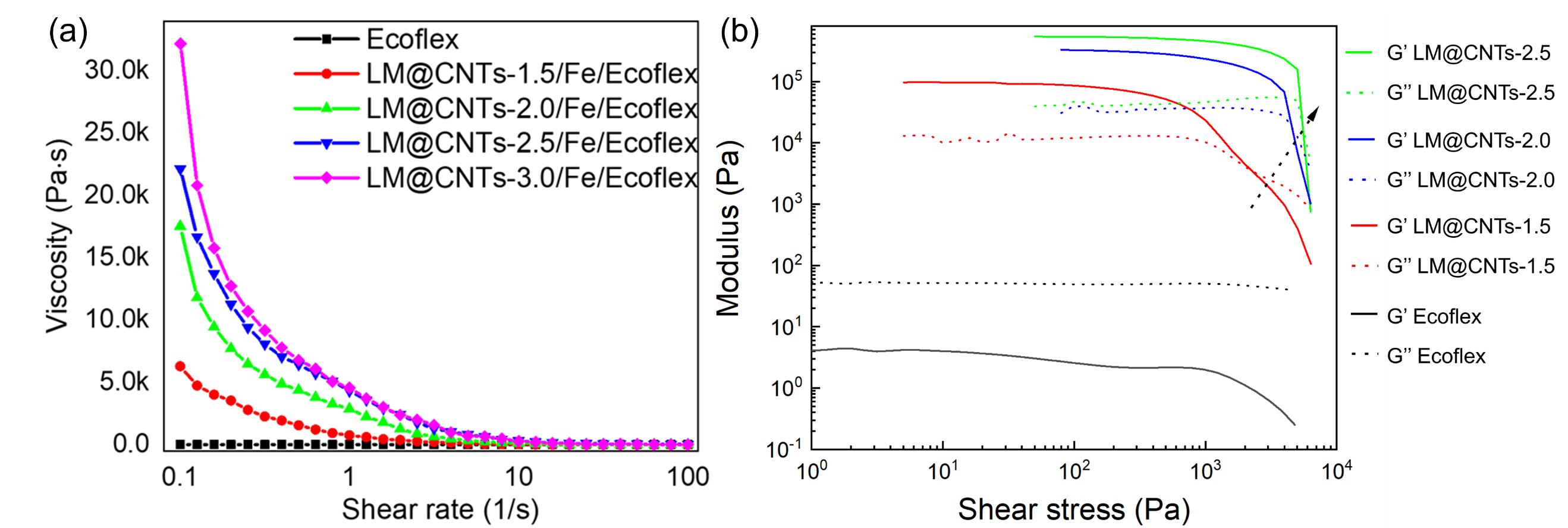
**

**Figure S3. The rheological behavior of Ecoflex and LM@CNTs-n/Fe/Ecoflex (n from 1.5 to 2.5/3.0)**. (a) Viscosity of inks with varying CNTs under different shear rate and (b) storage modulus (G’) and loss modulus (G’’) of inks with varying CNTs content under different shear stress at 1 Hz.


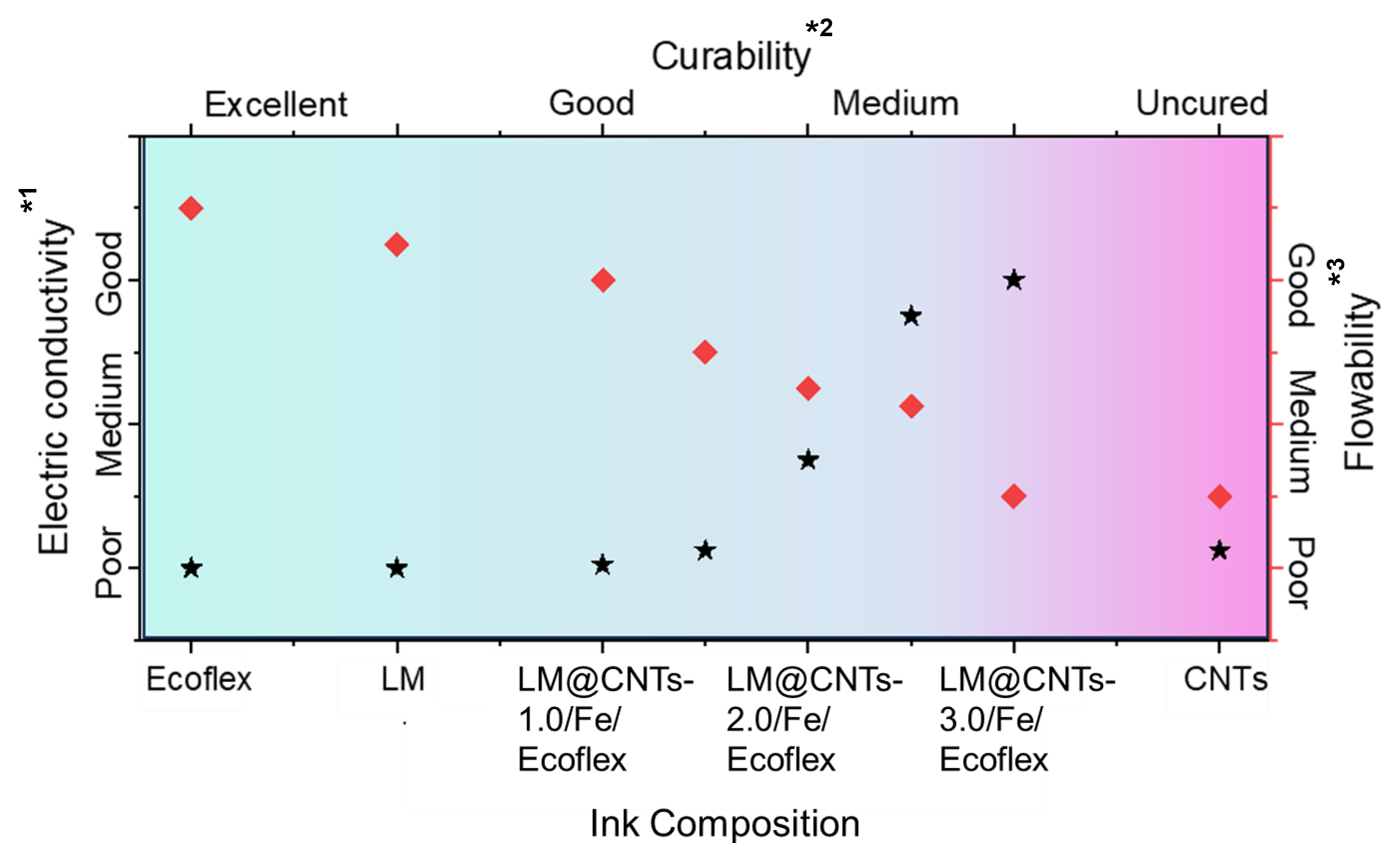


**Figure S4.** The qualitative presentation of electric conductivity, curability and flowability of ink with different compositions. *1. Poor: > 2000 kΩ·cm; Medium: in the range from 500 kΩ·cm to 2000 kΩ·cm; Good: < 500 kΩ·cm. *2. Excellent: Completely cured within 1 h at 80 °C; Good: Completely cured within 1 h to 3 h at 80 °C; Medium: Completely cured within 3 h to 24 h; Uncured: Not completely cured within 24 h. *3. Good: < 6000 Pa·s; Medium: 6000 Pa·s to 30000 Pa·s; Poor: > 30000 Pa·s. (Shear rate = 0.1 s^-1^, Temperature = 25 °C)


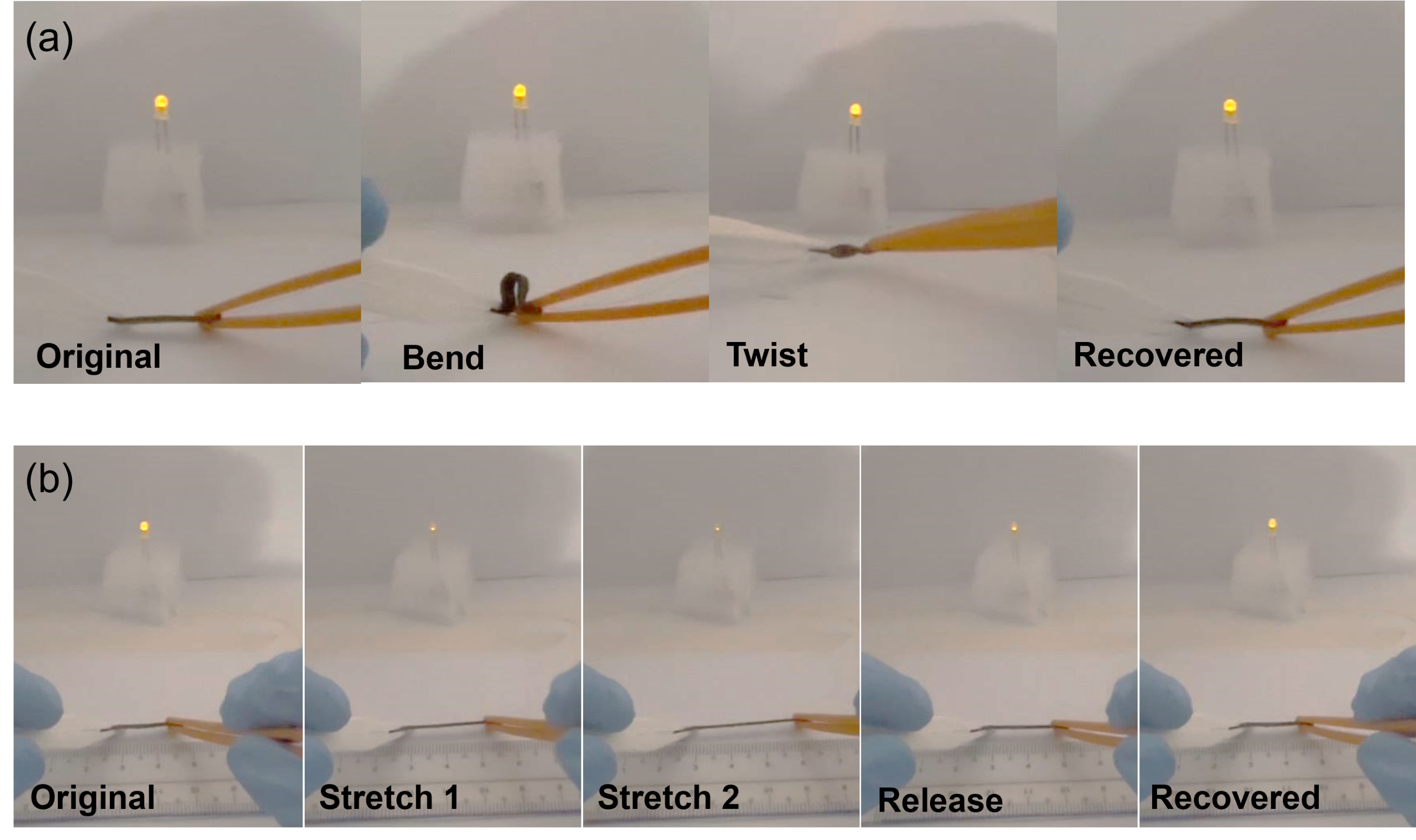


**Figure S5.** Qualitative resistance test for (a) sheet LM@CNTs-2.5/Fe/Ecoflex sample with an original resistance of 150 kΩ under bending and twisting and (b) linear LM@CNTs-2.5/Fe/Ecoflex sample with an original resistance of 200 kΩ under stretching. The term "recovered" here refers to the state that sensor regaining its nearly original shape after experiencing deformation, once external forces are removed.


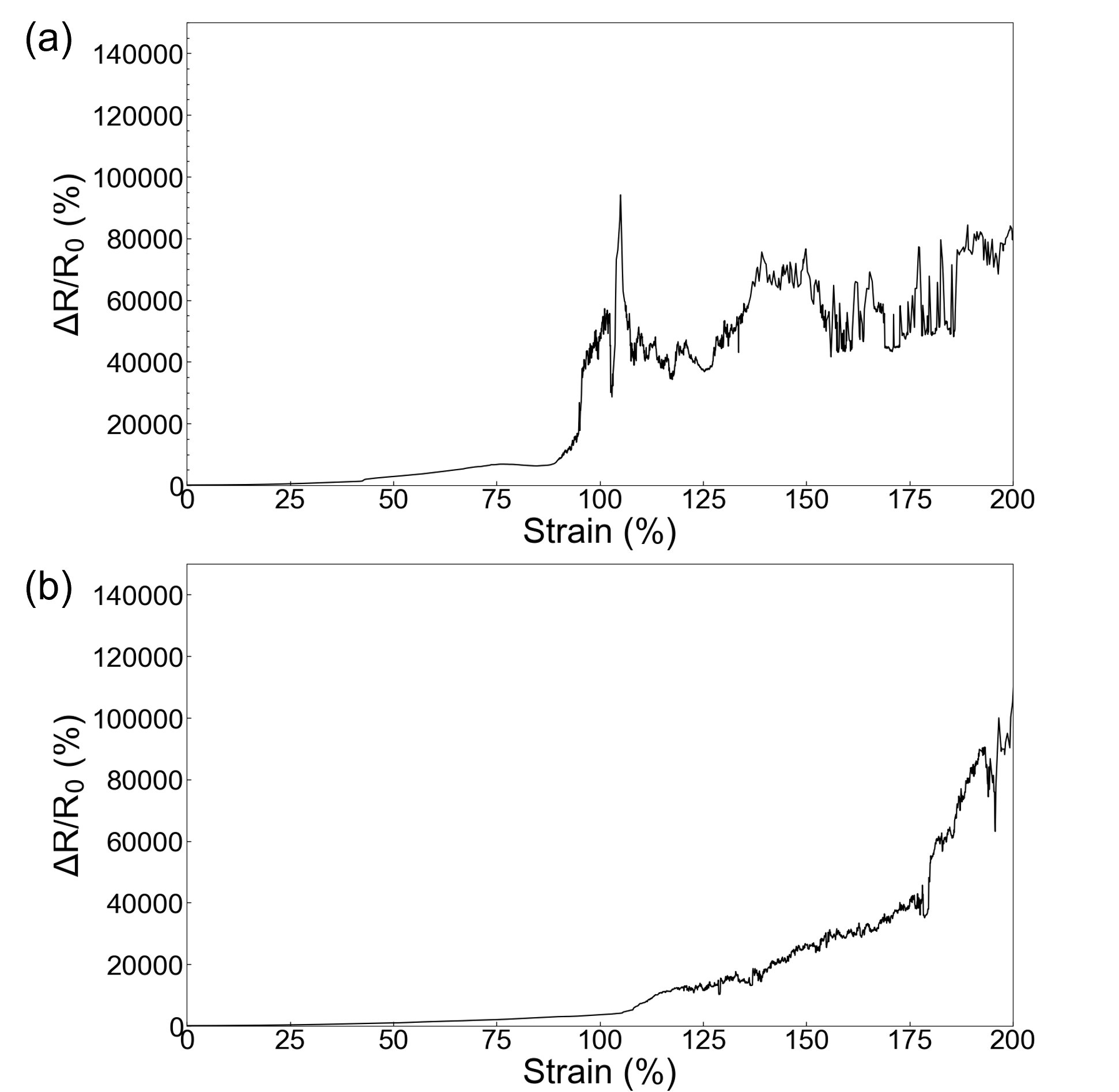


**Figure S6.** Relative resistance-strain curve for (a) LM@CNTs-1.5/Fe/Ecoflex sensor and (b) LM@CNTs-2.0/Fe/Ecoflex sensor.
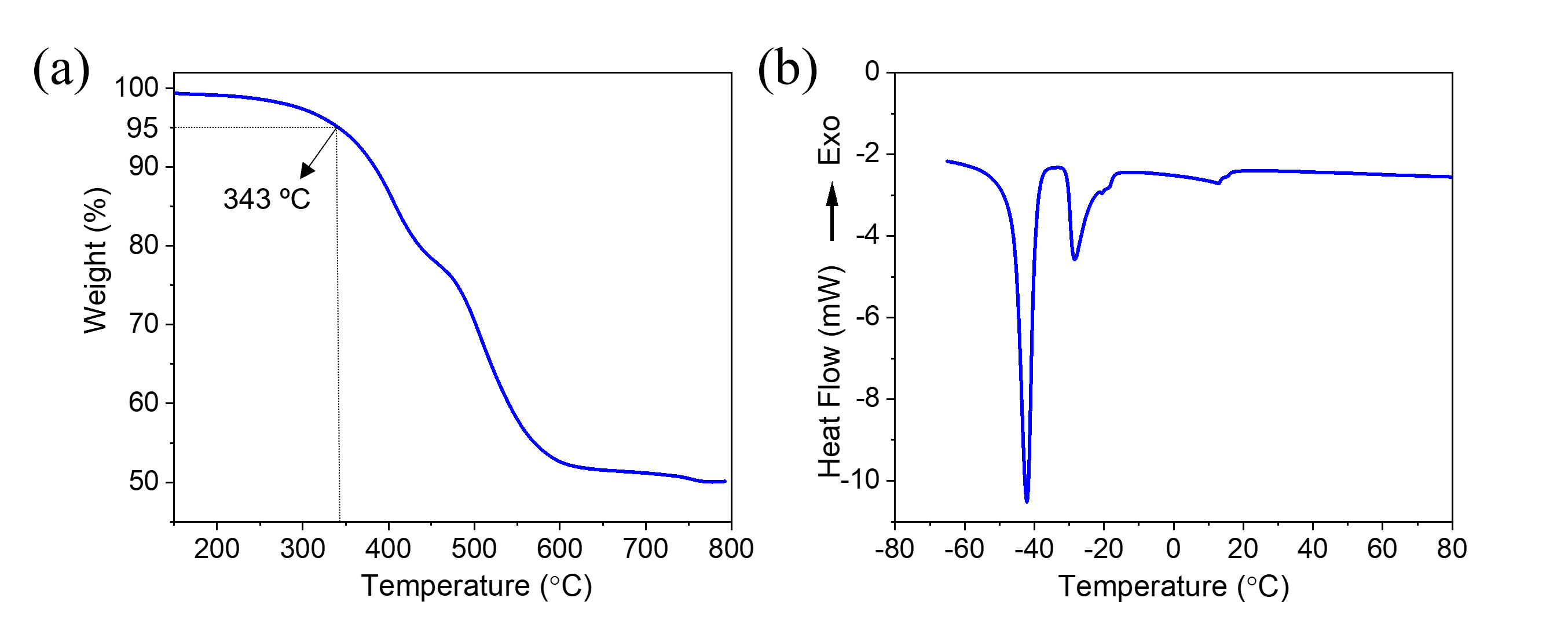


**Figure S7. The thermal properties of LM/Ecoflex sample.** (a) The TGA curve and (b) the DSC curve.


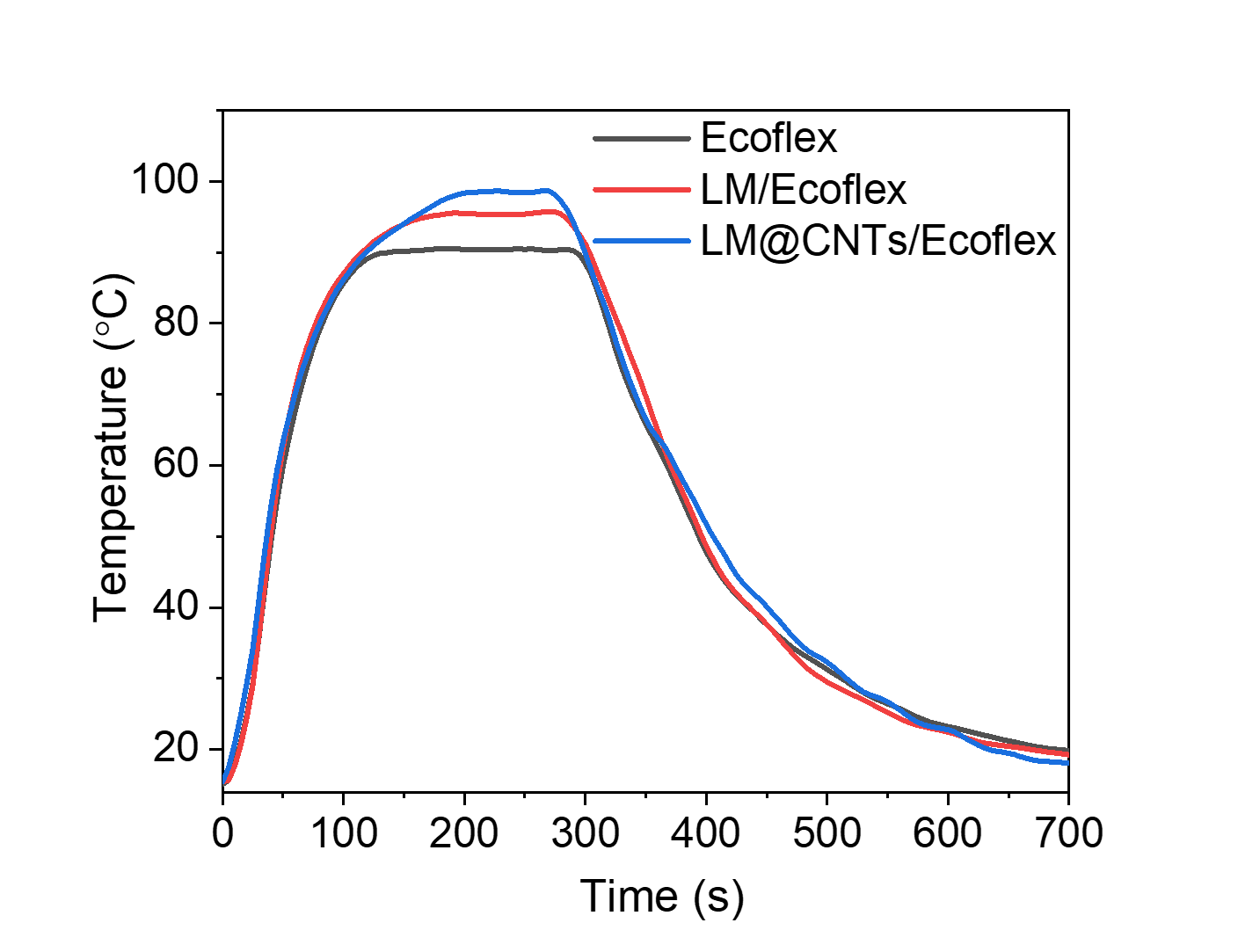


**Figure S8.** Temperature/time curves for Ecoflex, LM/Ecoflex and LM@CNTs-2.5/Fe/Ecoflex heated on hot plate at 120 ºC and then cooled in air.


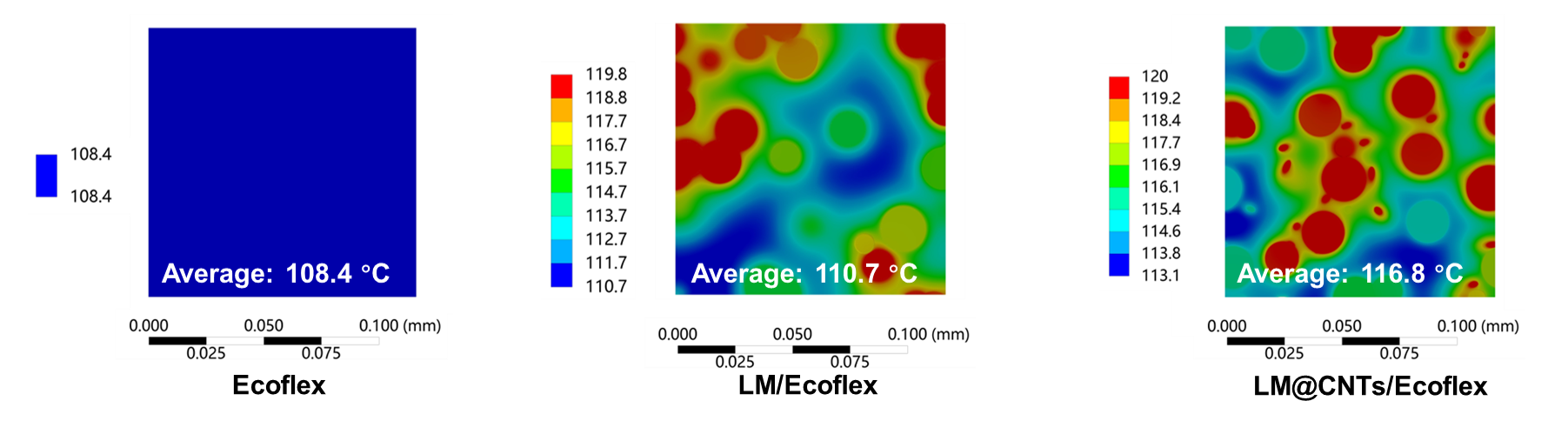


**Figure S9.** Simulated thermal equilibrium in the upper surface of thin (106 μm × 106 μm × 21 μm) sheets of different compositions on a heating stage at 120 ºC.

**Table S1.** Simulation details.

| Sheet | Geometry | 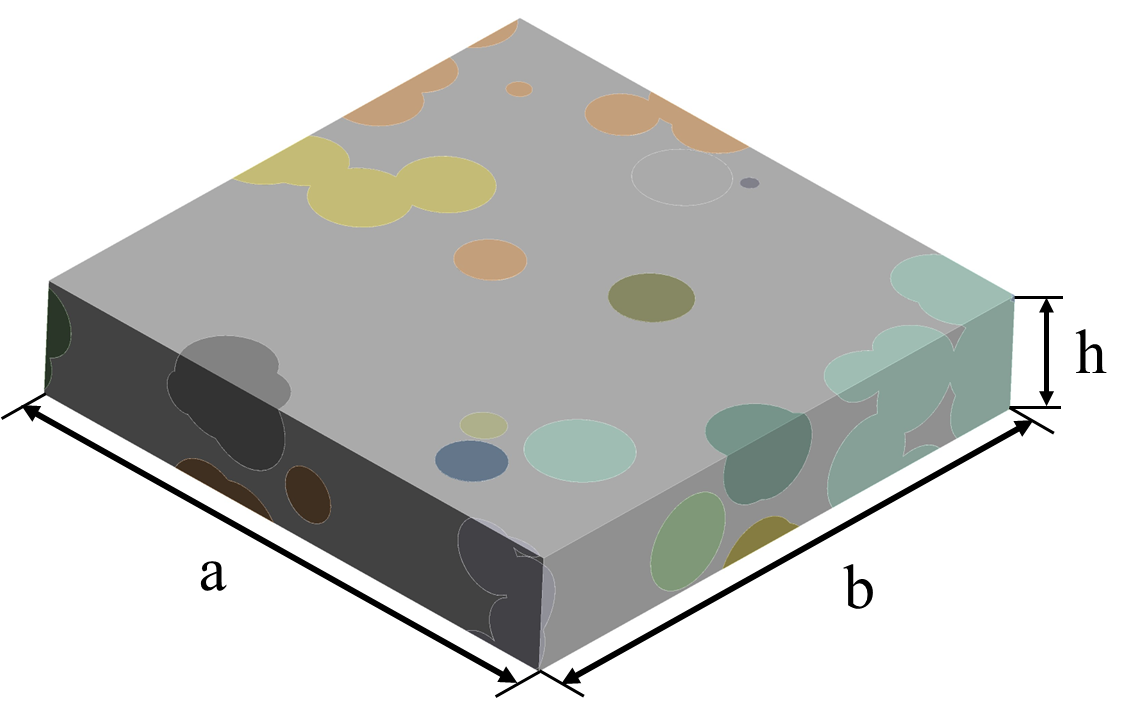 |
| --- | --- | --- |
|  |  | a = b = 106 μm, h = 21 μm |
| Inclusions | Inclusion Volume Fraction | LM: 0.236 (if any) |
|  |  | CNTs: 0.011 (if any) |
|  |  | Ecoflex: Bal. |
|  | Inclusion Shape | LM: Spherical |
|  |  | CNTs: Curved cylindrical (aspect ratio = 200) |
|  | Inclusion Orientation | Random |
| Thermal Properties | Thermal Conductivity  (Wm^-1^K^-1^) | LM: 23.2^[1]^ |
|  |  | CNTs: 4000^[2]^ |
|  |  | Ecoflex: 0.16^[3,4]^ |
|  | Convective Heat Transfer Coefficient (Wm^-2^K^-1^) | 1000* |
| Boundary Conditions | 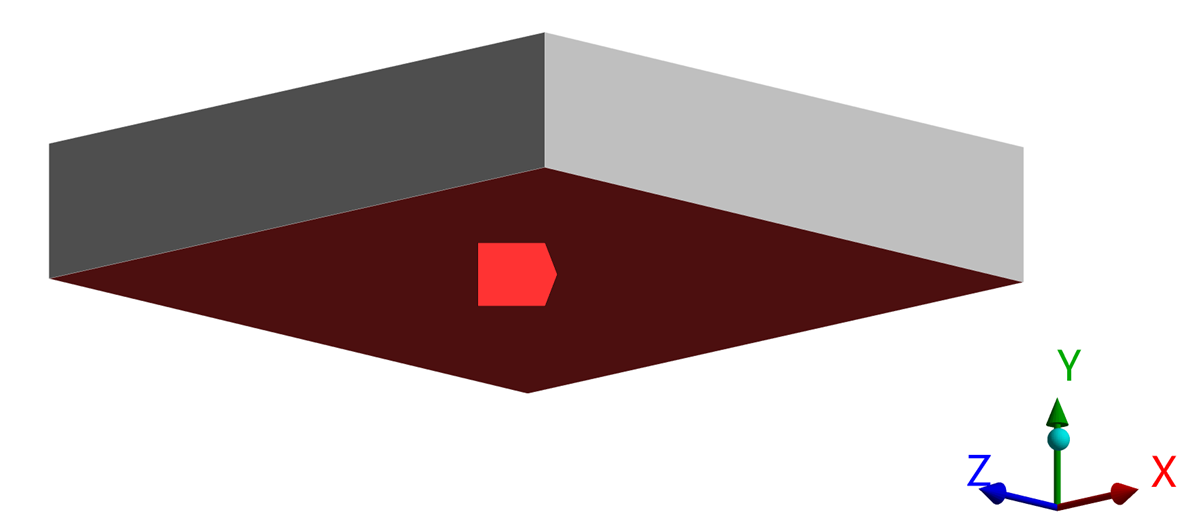  Bottom Face | Temperature = 120 ºC |
|  | 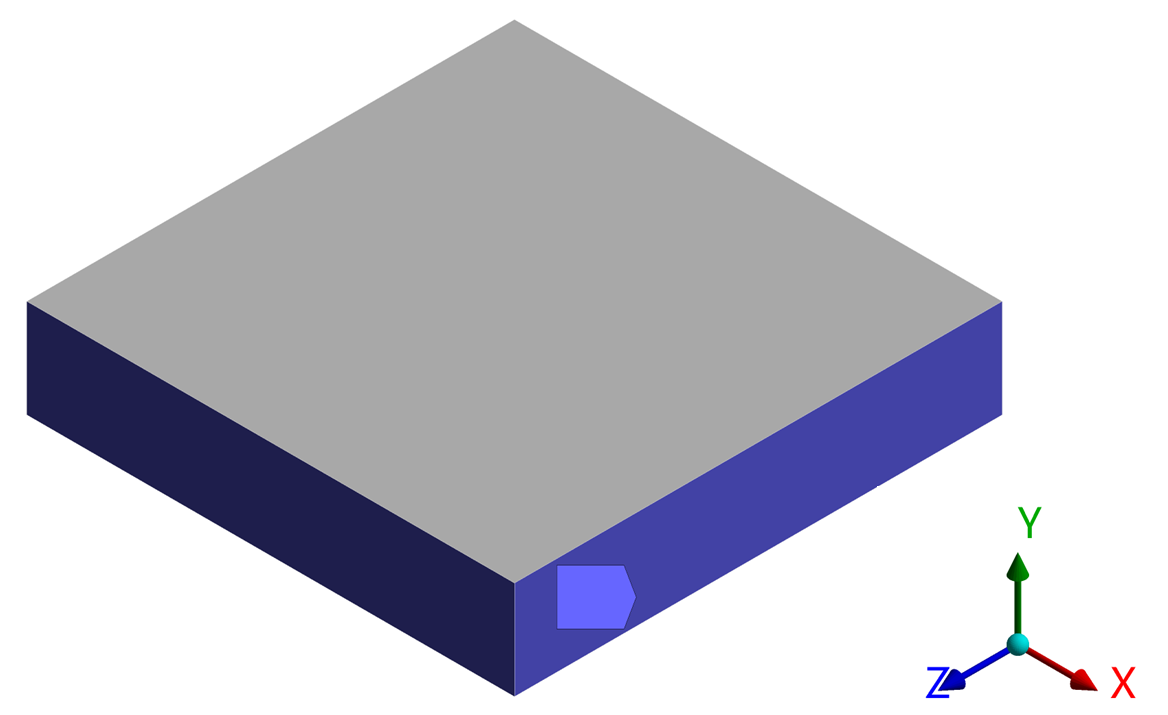  Side Faces | Adiabatic |
|  | 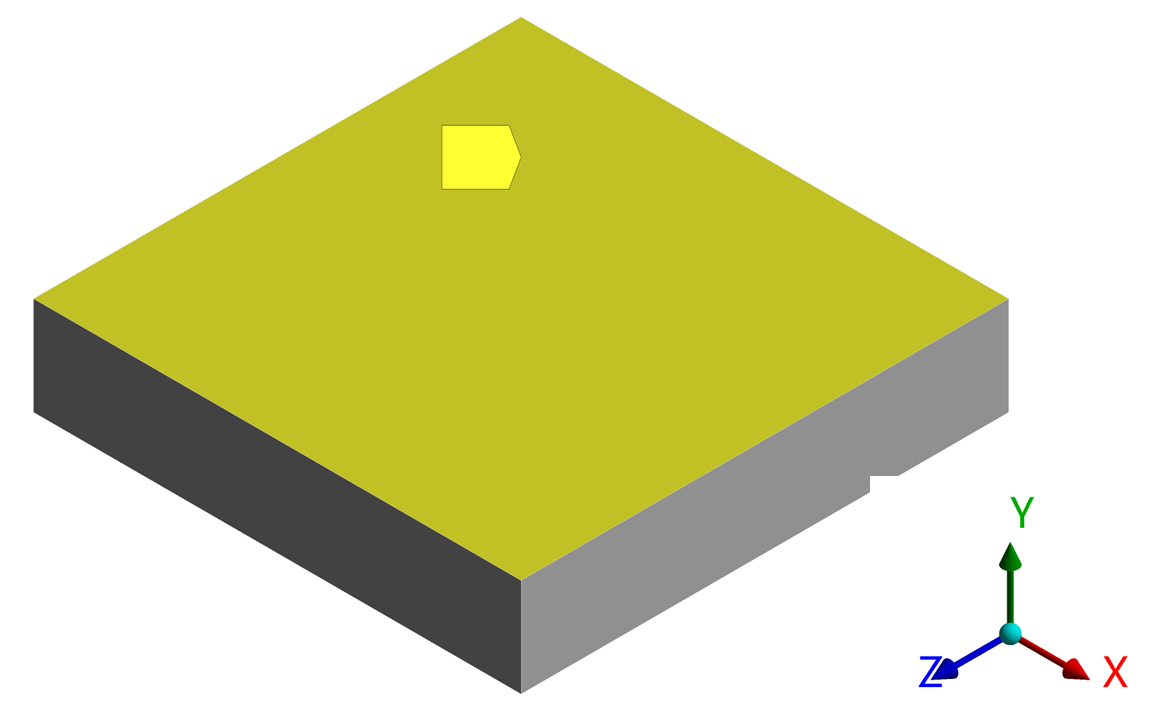  Top Face | Convection (1000 W m^-2^ K^-1^)  (Ambient temperature = 22 ºC) |

*. Considering the small sheet dimension, the temperature variation under a typical convective heat transfer coefficient (20 W m^-2^ K^-1^)^[5]^ is minimal. Consequently, for this simulation, a value of 1000 W m^-2^ K^-1^ was adopted to more effectively highlight the distinctions between different materials.

**References**

1. S. Ki, J. Shim, S. Oh, E. Koh, D. Seo, S. Ryu, J. Kim, Y. Nam, *Int. J. Heat Mass Transf.* **2021**, *170*.

2. Z. Han, A. Fina, *Prog. Polym. Sci. (Oxford)* **2011**, *36*, 914.

3. X. D. Zhang, Z. T. Zhang, H. Z. Wang, B. Y. Cao, *ACS Appl. Mater. Interfaces* **2022**.

4. J. Choi, C. Dun, C. Forsythe, M. P. Gordon, J. J. Urban, *J. Mater. Chem. A Mater.* **2021**, *9*, 15696.

5. R. Conti, A. A. Gallitto, E. Fiordilino, *arXiv preprint* **2014**.
